# Rechallenge capecitabine after fluoropyrimidine-induced cardiotoxicity in rectal cancer: A case report

**DOI:** 10.1097/MD.0000000000014057

**Published:** 2019-01-11

**Authors:** Tao Peng, Yulu Ouyang, Kanger Tong

**Affiliations:** Department of Oncology, Hospital of Traditional Chinese Medicine, Keqiao District, Shaoxing City, Zhejiang Province, China.

**Keywords:** capecitabine, cardiotoxicity, fluoropyrimidine

## Abstract

**Rationale:**

Fluoropyrimidine-induced cardiotoxicity is a rare but potentially serious toxicity. The most common symptom is anginal chest pain.

**Patient concerns:**

A 35-year-old woman was diagnosed with rectal cancer with metastasis to the liver.

**Diagnosis:**

A computed tomography scan showed a 9.3 × 4.5-cm predominantly hypodense lesion within the left lobe of the liver and thickening of the rectum. Liver biopsy showed moderately differentiated adenocarcinoma with necrosis involving the liver parenchyma, and immunohistochemistry for mismatch repair proteins indicated that the tumor was positive for MutL Homolog 1, MutS Homolog 2, MutS Homolog 6, and Protein Homolog 2. Rectal biopsy indicated moderately differentiated adenocarcinoma.

**Interventions:**

She received chemotherapy of fluorouracil 1600 mg/m^2^, leucovorin 500 mg/m^2^, and irinotecan 100 mg/m^2^ every week. During the second cycle of chemotherapy, she developed severe anginal chest pain. We replaced fluorouracil with capecitabine 1500 mg (3 pills) a day every 2 weeks, with 1 week off, with irinotecan 100 mg/m^2^ on day 1 and bevacizumab 5 mg/kg at 200 ml/h for 30 min every 2 weeks. She was treated with chemotherapy for approximately 6 months.

**Outcomes:**

The liver lesion showed a significant response to chemotherapy, so she underwent resection of the liver tumor and rectum. After the surgery, she received radiation therapy to the rectal area, and 3 months of chemotherapy were administered prior to colostomy reversal.

**Lessons:**

Although the mechanism of fluoropyrimidine-induced cardiotoxicity is still uncertain, our case provides clinical evidence that cardiotoxicity could be a dose-related complication. Reducing the dose of fluoropyrimidine should be considered as a strategy after fluoropyrimidine-induced cardiotoxicity. However, this must be discussed with a multidisciplinary team including oncologists and cardiologists. Close monitoring of serial biomarkers and echocardiography are necessary for early diagnosis of cardiotoxicity.

## 1 Introduction

Fluoropyrimidines are common chemotherapies, including drugs such as 5-fluorouracil (5-FU) and capecitabine. Since its discovery, 5-FU has become a standard chemotherapy for many solid tumors. It is commonly administered in gastrointestinal carcinoma and many other adenocarcinomas and squamous cell carcinomas.^[1]^ Capecitabine is an oral prodrug of 5-FU that is converted to 5-FU through a three-step enzymatic cascade. Therefore, capecitabine is also widely used in multiple types of carcinomas. The side effects of fluoropyrimidines include nausea, emesis, diarrhea, myelosuppression, and hand-foot syndrome. In addition, fluoropyrimidine-induced cardiotoxicity is a rare but potentially serious toxicity.^[2–5]^ The most common symptom of cardiotoxicity is anginal chest pain, and other symptoms include dyspnea and hypertension. Severe heart failure due to 5-FU-induced cardiotoxicity has also been reported.^[6]^ The likely mechanisms of fluoropyrimidine-induced cardiotoxicity include coronary vasospasm and direct cytotoxicity. When 5-FU-induced cardiotoxicity occurs, discontinuation of 5-FU usually relieves symptoms within hours. However, there are no certain guidelines as to how to proceed with treatment; whether to attempt capecitabine or abandon fluoropyrimidines altogether is still contentious. Here we present a 35-year-old woman who was successfully treated with a reduced dose of capecitabine after fluoropyrimidine-induced cardiotoxicity.

## 2 Case report

**Figure 1:**
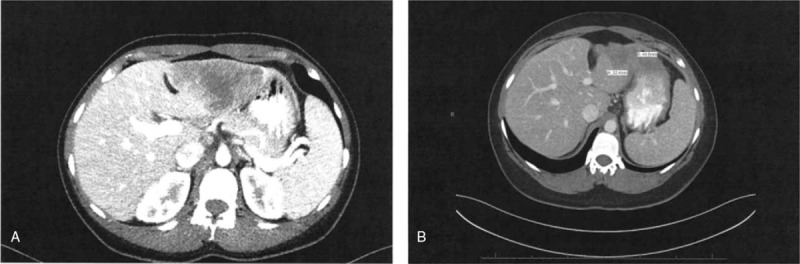
A. Abdominal computed tomography (CT) in July 2015 showed a 9.3 × 4.5 cm liver mass; B. Abdominal CT in April 2016 showed that the liver mass had reduced in size to 3.2 × 4.5 cm.

A 35-year-old woman was admitted to the emergency room of our institution because of hematochezia in July 2015. Computed tomography (CT) showed a 9.3 × 4.5-cm predominantly hypodense lesion within the left lobe of the liver (Fig. 1 A) and thickening of the rectum. There was no definite evidence of metastatic disease in the chest. Pathology of the liver biopsy specimen indicated moderately differentiated adenocarcinoma with necrosis involving the liver parenchyma, and immunohistochemistry for mismatch repair proteins was positive for MutL Homolog 1, MutS Homolog 2, MutS Homolog 6, and Protein Homolog 2. Rectal biopsy indicated moderately differentiated adenocarcinoma. Therefore, she was diagnosed with rectal cancer with metastasis to the liver. The patient provided consent for treatment, and she was administered fluorouracil 1600 mg/m^2^, leucovorin 500 mg/m^2^, and irinotecan 100 mg/m^2^ every week.

She experienced pain in her upper arm and back after the first round of chemotherapy. After the second round of chemotherapy, she had developed severe anginal chest pain, with ST elevations on electrocardiography, and we discontinued chemotherapy. Because of the chest pain, we replaced the fluorouracil with capecitabine. However, any dose higher than 1500 mg (3 pills) a day caused anginal chest pain and shoulder ache. Therefore, we reduced the dose of capecitabine to 1500 mg (3 pills) a day every 2 weeks, with 1 week off, with irinotecan 100 mg/m^2^ on day 1 and bevacizumab 5 mg/kg at 200 ml/h for 30 min every 2 weeks. She received chemotherapy for approximately 6 months and experienced no further chest pain.

**Figure 2:**
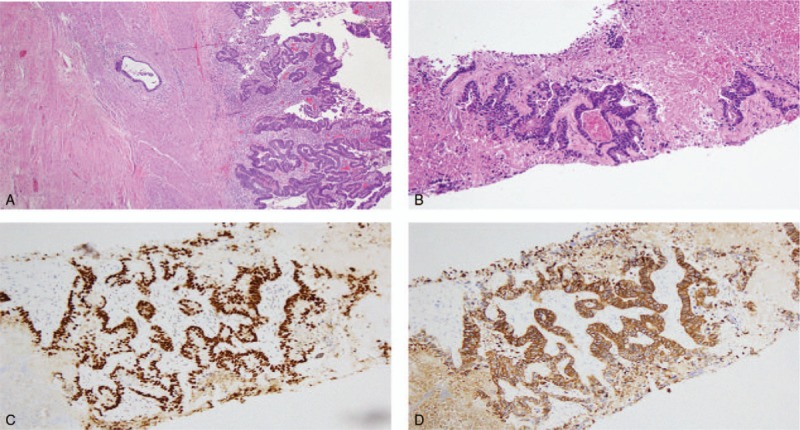
A. Moderately differentiated adenocarcinoma of the sigmoid colon (hematoxylin-eosin [H&E], 4×); B. Metastatic adenocarcinoma of the liver (H&E, liver biopsy, 10×); C. Positive staining for caudal-related homeobox transcription factor 2 in the tumor cells (liver biopsy, 10×); D. Positive staining for cytokeratin 20 in the tumor cells (liver biopsy, 10×).

The liver lesion showed a significant response to chemotherapy. CT on April 27, 2016 showed an ill-defined heterogeneous lesion with dystrophic calcification in segment 3 of the liver associated with capsular retraction measuring approximately 3.2 × 4.5 cm (down from 9.3 × 4.5 cm since July 2015) (Fig. 1). Moreover, the CT also showed focal thickening of the rectosigmoid junction with multiple surrounding subcentimeter lymph nodes. In July 2016, she underwent liver tumor and rectal resection. The pathology indicated a moderately differentiated adenocarcinoma, 2.3 cm in size, extensively invading the perirectal adipose tissue (American Joint Committee on Cancer, 7th Edition classification: ypT3N2aM1a). Immunohistochemistry indicated the tumor was positive for caudal-related homeobox transcription factor 2 and cytokeratin 20 expression (Fig. 2). There were metastases in 5 of 21 lymph nodes. She received radiation therapy to the rectal area, and based on the pathology, we administered 3 months of chemotherapy prior to the colostomy reversal. She received the same modified regimen and did not experience any further chest pain.

## 3 Discussion

Cardiotoxicity is a potentially lethal complication of fluoropyrimidine treatment. With the widespread use of fluoropyrimidines and the lack of knowledge about this complication or subclinical cardiotoxicity, fluoropyrimidine-induced cardiotoxicity may be more common in the clinic that is documented in the literature. It is difficult to tell whether 5-FU or capecitabine is more likely to induce cardiotoxicity. A systematic review by Polk et al found that, in larger studies, the incidence of cardiotoxicity with 5-FU was lower (1.2%–4.3%) than that with capecitabine (3%–35%).^[7]^ However, an analysis including metastatic colorectal cancer (n = 1189) and metastatic breast cancer (n = 236) found that the incidence of cardiotoxicity in patients receiving capecitabine monotherapy was within the range of that with 5-FU.^[8]^

The exact mechanism of fluoropyrimidine-induced cardiotoxicity is still unknown, although coronary vasospasm seems to be the most accepted mechanism.^[1,2,4,9]^ Mosseri et al demonstrated that 5-FU causes direct, endothelium-independent vasoconstriction of the vascular smooth muscle in rabbit aortic rings.^[10]^ Based on this mechanism, 5-FU may be administered in combination with vasodilators to reduce the incidence of cardiotoxicity. However, the use of prophylactic vasodilators to prevent vasospasm did not help in some clinical reports, suggesting that cardiotoxicity depends on multiple factors. Other studies have postulated mechanisms including direct cellular toxicity, release of endothelin-1, and a neural-mediated mechanism involving the adrenergic nervous system. Additionally, myocardial injury, thrombogenic effects, immune-allergic reaction, and ischemia secondary to coronary artery spasm have all been implicated in the mechanism of fluoropyrimidine-induced cardiotoxicity.^[4]^

The most common symptom of cardiotoxicity is anginal chest pain. Other symptoms include dyspnea, hypertension, and myocardial infarction. When cardiotoxicity is suspected, early recognition is crucial to prevent the progression of toxicity. Anginal chest pain usually occurs 2 to 5 days after the administration of 5-FU,^[11]^ whereas the average time to cardiotoxicity occurrence with capecitabine treatment is 4 days (range of 2–15 days).^[12]^ If the patient experiences anginal chest pain that is suspected to be fluoropyrimidine-induced cardiotoxicity, discontinuing the fluoropyrimidine will usually relieve the chest pain within hours. Prompt electrocardiography should be performed to assess ischemic ST changes, conduction abnormalities, and arrhythmias. Echocardiography, cardiac biomarkers like troponin and brain natriuretic peptide, and coronary angiography are recommended to assist diagnosis.^[1]^ However, the ideal treatment strategy following fluoropyrimidine-induced cardiotoxicity remains unclear. Once cardiotoxicity has occurred, the safety of a rechallenge with a modified 5-FU- or capecitabine-based regimen is uncertain, and no widely accepted recommendations have been released.^[13]^ Saneeymehri et al rechallenged capecitabine with a patient who experienced chest pain with 5-FU administration. The cardiac symptoms subsided after initiation of capecitabine and the patient tolerated the treatment well.^[3]^ If chest pain occurs with capecitabine treatment, fluoropyrimidines will usually not be further considered. Golias et al. reported a 46-year-old woman with gastric sarcoma who was administered cisplatin after capecitabine-induced cardiotoxicity.^[2]^ In our case, we rechallenged capecitabine after 5-FU-induced cardiotoxicity. However, our patient still experienced chest pain with a high dose of capecitabine. We attempted a low dose of capecitabine of 1500 mg per day, and it was well tolerated.

## 4 Conclusions

Fluoropyrimidine-induced cardiotoxicity may be more common than is currently thought. Because of its potentially serious toxicity, doctors and patients should be more aware of this potential toxicity. However, given the promising antitumoral efficacy of fluoropyrimidines, their use should not be discouraged. Although the mechanism of fluoropyrimidine-induced cardiotoxicity is still uncertain, our case provides clinical evidence that it could be a dose-related complication. Reducing the dose of fluoropyrimidine may be considered as a strategy after fluoropyrimidine-induced cardiotoxicity, although this should first be discussed with the oncologist and cardiologist, and close monitoring of serial biomarkers and echocardiography are necessary for the early diagnosis of cardiotoxicity. More evidence based on multicenter clinical trials is needed to determine the ideal course for fluoropyrimidine-induced cardiotoxicity.

## Author contributions

**Methodology:** Kanger Tong.

**Writing – original draft:** Tao Peng.

**Writing – review & editing:** Yulu Ouyang.
